# Seasonal Dynamics of Atlantic Herring (*Clupea harengus* L.) Populations Spawning in the Vicinity of Marginal Habitats

**DOI:** 10.1371/journal.pone.0111985

**Published:** 2014-11-05

**Authors:** Florian Eggers, Aril Slotte, Lísa Anne Libungan, Arne Johannessen, Cecilie Kvamme, Even Moland, Esben M. Olsen, Richard D. M. Nash

**Affiliations:** 1 Institute of Marine Research, Bergen, Norway; 2 Department of Biology, University of Bergen, Bergen, Norway; 3 Department of Life and Environmental Sciences, University of Iceland, Reykjavík, Iceland; 4 Institute of Marine Research, Flødevigen, Norway; 5 Centre for Ecological and Evolutionary Synthesis (CEES), Department of Biosciences, University of Oslo, Oslo, Norway; 6 Department of Natural Sciences, Faculty of Science and Engineering, University of Agder, Kristiansand, Norway; Technical University of Denmark, Denmark

## Abstract

Gillnet sampling and analyses of otolith shape, vertebral count and growth indicated the presence of three putative Atlantic herring (*Clupea harengus* L.) populations mixing together over the spawning season February–June inside and outside an inland brackish water lake (Landvikvannet) in southern Norway. Peak spawning of oceanic Norwegian spring spawners and coastal Skagerrak spring spawners occurred in March–April with small proportions of spawners entering the lake. In comparison, spawning of Landvik herring peaked in May–June with high proportions found inside the lake, which could be explained by local adaptations to the environmental conditions and seasonal changes of this marginal habitat. The 1.85 km^2^ lake was characterized by oxygen depletion occurring between 2.5 and 5 m depth between March and June. This was followed by changes in salinity from 1–7‰ in the 0–1 m surface layer to levels of 20–25‰ deeper than 10 m. In comparison, outside the 3 km long narrow channel connecting the lake with the neighboring fjord, no anoxic conditions were found. Here salinity in the surface layer increased over the season from 10 to 25‰, whereas deeper than 5 m it was stable at around 35‰. Temperature at 0–5 m depth increased significantly over the season in both habitats, from 7 to 14°C outside and 5 to 17°C inside the lake. Despite differences in peak spawning and utilization of the lake habitat between the three putative populations, there was an apparent temporal and spatial overlap in spawning stages suggesting potential interbreeding in accordance with the metapopulation concept.

## Introduction

Typically, fish species may be split into populations based on their degree of reproductive isolation from each other in space and/or time, which could be reflected in genetic or phenotypic differences driven by diverging environmental conditions [1]–[3]. Under such circumstances exploitation on one population should have little effect on the population dynamics of a neighboring population, and therefore it is also common to assess and manage such populations separately [4], [5]. On the other hand, there are also examples where populations are recognized to be separate with diverging spawning season and/or spawning area, but due to mixing in other seasons a separate management of the populations may be difficult [6], [7]. The need to identify the different populations, especially where exploitation occurs on mixtures of populations is important for successful management [8], [9]. Fisheries biologists therefore often use the term stock instead of population in their fisheries advice; i.e. sometimes a population is harvested and therefore managed as one stock and at other times several separate populations are harvested and managed as one stock. In Begg et al. [10] the concept of a fish stock was simply defined as characteristics of semi-discrete groups of fish with some definable attributes, which are of interest to fishery managers. The definition of ICES [11] for a stock as a part of a fish population usually with a particular migration pattern, specific spawning grounds, and subject to a distinct fishery, will be used hereby. In theory, all individual fish in an area, being part of the same reproductive process, are comprised as a stock. When referring to fisheries management, the term “stock” is used, otherwise the term “population” is preferred.

Atlantic herring (*Clupea harengus* L.) is characterized by highly complex population structure and migration patterns [12]. It is an iteroparous clupeid, becoming sexually mature at two or three years of age, and a total spawner that aggregates at spawning, laying benthic eggs on shells, gravel, coarse sand and small stones at depths down to 250 m [13]. The larvae hatch after 2–4 weeks depending on temperature [14], [15]. They drift with currents until metamorphosis [16]–[18], with vertical migration increasing throughout ontogeny [19], [20] and affecting the dispersal trajectories of larvae. The different herring populations are generally classified according to their spawning grounds, which, due to the specific spawning substratum requirements, are fixed geographically and used at a predictable time of the year. Due to physical and geographical barriers, such as prevailing currents and general location of nursery areas, there is often little mixing of larvae, thus tending to isolate the different populations. However, there are occasions where larvae and juveniles may co-occur. Under these circumstances identification of individuals or groups of individuals is undertaken using otolith or meristic characters [1], [21]–[24] as well as genetic markers [25]–[28]. In the 1950–60s experimental studies [29]–[31] demonstrated that myotome counts in herring were influenced by both temperature (negatively) and salinity (positively) experienced during the incubation period. The consequence is that mean vertebral count of adult herring is an indicator of spawning ground and spawning times and in some cases also population.

In Norwegian waters some herring populations occupy marginal habitats along the coastline and deep inside fjords, most of which are thought to be stationary with adaptations to local conditions. Hence, they are often phenotypically and, in some occasions, genotypically different from the nearby oceanic population. Examples of such local herring populations are Trondheimsfjord herring [32], [33], Borge Poll herring [34], Lusterfjord herring [35], Lindåspollene herring [36], Balsfjord herring [37], Lake Rossfjord herring [38] and the summer/autumn spawners in northern Norway [39]. Despite the discovery of these local populations, the overall research effort targeting marginal areas along the Norwegian coast has been rather low, and it is therefore expected that a number of additional local populations may exist.

Migratory coastal or oceanic populations may occasionally enter the marginal habitats along the Norwegian coast and mix with local herring. This is in accordance with the metapopulation concept, where two or more distinguished subpopulations have variable but moderate interbreeding and significant gene flow [40]. Temporal and spatial overlap during spawning may allow genetic exchange between subpopulations, which is a prerequisite for the existence of metapopulations. An example of such an overlap was demonstrated by Johannessen et al. [41],[42] in the local Lindåspollene herring, where significant changes in life history traits over a 50 year period were linked to genetic exchange with the oceanic population according to the metapopulation concept.

An important mixing area for herring is the northeastern North Sea and Skagerrak, where three different stocks may occur, Norwegian Spring Spawners (NSS), North Sea Autumn Spawners (NSAS) and Western Baltic Spring Spawners (WBSS). Some of these stocks comprise different herring populations, such as coastal Skagerrak spring spawners or more local herring populations, which are not directly subjected to a distinct fishery. The different populations (stocks) can be distinguished by spawning site, spawning season, meristic characters such as the number of vertebrae (VS) and otolith characteristics [23], [41].

Of particular interest in the Skagerrak area is a brackish water environment inside Landvikvannet, an inland lake in southern Norway connected to the open sea through an artificial channel. The Institute of Marine Research (IMR) has been sampling herring in Landvikvannet on regular basis since 1984, mainly in May. Data from these investigations demonstrate that herring inside the lake are normally ripe or with running gonads, with a low mean vertebral number (<56.0), slow growth and high fecundity [43], [44]. This has led to the hypothesis that the lake is visited on an annual basis by a herring population with specific adaptations to spawning in these brackish water environments. However, in the coastal areas outside the lake, ripe and spawning herring with higher growth and mean vertebral numbers (56.0–57.5) have occurred in samples over the period February–June [43]. This indicates that there may be a mixture of several populations in the area with some temporal and spatial overlap in spawning, which could be linked to spatial seasonal differences in environmental conditions. Such metapopulation dynamics may be revealed by a more detailed seasonal sampling outside the May period normally focused on in IMR's investigations in Landvikvannet. Hence, the principal objective of the present study was to explore the overlap in time, space and maturation stages of phenotypically different herring appearing in Landvikvannet and neighboring fjord areas and their dependence on seasonal changes in environmental conditions.

## Material and Methods

### Study area

**Figure 1:**
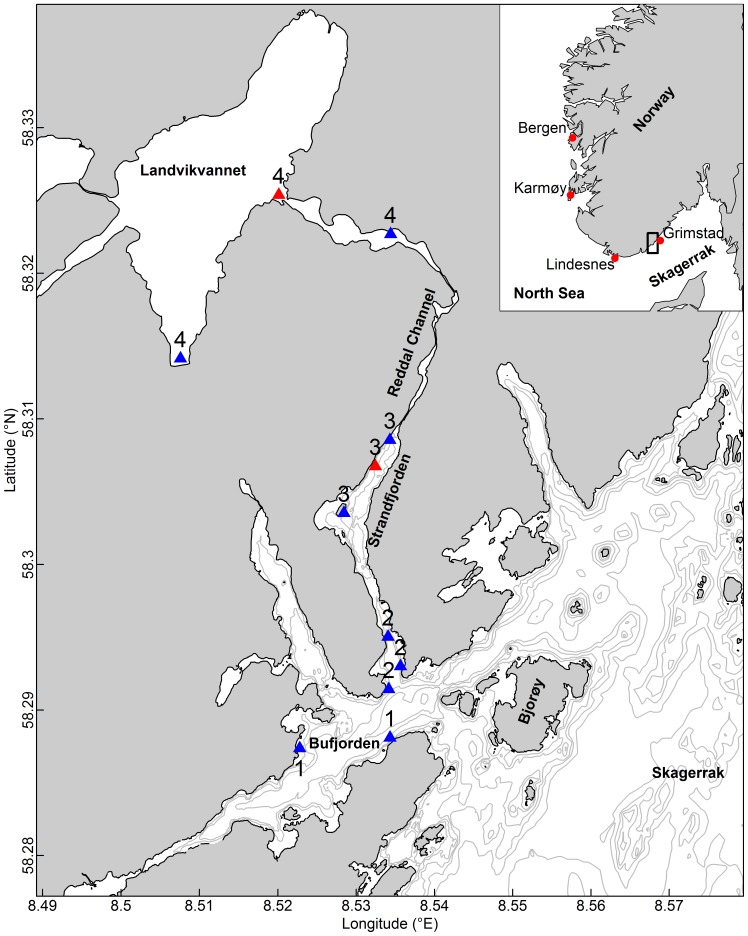
Map of the study area. The map shows CTD-stations (red) and gillnet stations (blue) in 1 = Bufjorden, 2 = Outer part of Strandfjorden, 3 = Inner part of Strandfjorden, 4 = Landvikvannet.

Landvikvannet is a 1.85 km^2^ lake located on the Norwegian Skagerrak coast (Figure 1). In 1877 a 3 km long channel (Reddal channel, Figure 1) was constructed, connecting the lake to the open sea. This narrow 1–4 m deep channel transformed Landvikvannet into a brackish system and in addition lowered the water level in the lake by 3 m. At the entrance of the lake there is a small 25 m deep basin. Further into the lake the bottom depth decreases rapidly to 7–10 m. Most of the shoreline is covered by reeds; otherwise the shore is rocky and steep. There is inflow of saltwater over the tidal cycle, whereas freshwater empties into the lake from streams, resulting in a halocline. Oxygen is depleted in the lower layers whereas the surface layer is oxygen rich. In Landvikvannet, herring have been caught by floating gillnets together with trout (*Salmo trutta*) and other freshwater fish since shortly after the channel was opened.

The Reddal channel drains into Strandfjorden (Figure 1), where conditions are estuarine. The outer Strandfjorden is narrow and shallow (1–7 m), whereas the inner part is deeper (10–13 m). Most herring samples were collected in the inner part, close to the mouth of the Reddal channel. The shore is rocky and steep with sparse macroalgae in the upper few meters. At depths >5–6 m the bottom consists of sand and mud. The outermost fjord (Bufjorden, Figure 1) is small with direct connection to Skagerrak. Strandfjorden is connected to the open ocean via Bufjorden (Figure 1). The entrance of Bufjorden is characterized by a 54 m deep basin. The physical environment is similar to Strandfjorden, only less influenced by fresh water runoff. Access to Bufjorden is from the south or east.

### Environmental data

To explore whether potential differences in habitat utilization and timing of peak spawning among herring populations were dependent on seasonal changes in environmental conditions, sampling of environmental data was undertaken between March and June 2012 both inside and outside the lake habitat. Note, that no stations could be sampled in February due to ice cover. Water samples were collected at the site where gillnets were moored in the inner part of Strandfjorden and at the entrance of Landvikvannet in the first basin (Figure 1). We measured temperature and salinity at depth with a CTD (STD/CTD – model SD204, SAIV Ltd. Environmental sensors and Systems, Bergen, Norway), while oxygen and hydrogensulfide concentrations were analyzed in the laboratory at the Institute of Marine Research (IMR). In the lake, water samples were collected each 0.5 meter down to the depth of oxygen depletion (hypoxic depth), which was found using the Winkler test [45], thereafter water samples were taken at 5 m depth intervals. The choice of position for sampling environmental data inside the lake is based on the depth contours of the area. The lake itself is rather shallow, and the bottom depth at most gillnet stations is 2–4 m. However, at the entrance the lake is at its deepest (25 m), which is why this position has been used since investigations started in the area in the 1980s. The environmental conditions at this site between 0 and 10 m have been examined thoroughly over a number of years and are comparable to conditions elsewhere in the lake and as such can be used to characterize the whole lake. These data are therefore representative of all gill net sampling sites.

### Biological data

To explore the potential overlap in time, space and maturation stages of phenotypically different herring appearing inside and outside the lake habitat, herring were sampled with gillnet over the full spawning season in 2012 (February–June) concurrently in both habitats (Figure 1, Table 1). In February, due to ice cover both in the lake and inner fjord habitats of Strandfjorden, samples were only taken further out in Bufjorden. The floating gillnets with a mesh size of 26 mm and 29 mm, a depth of 8 m and a length of approximately 10 m were used randomly in all areas. Soak time was 24 hours. This experiment was approved by the Norwegian committee for the use of animals in scientific experiments (FDU). Special permission to fish with floating gillnet inside Landvikvannet and in the connected fjord system in 2012 was given by the County Governor of Aust-Agder, Department of Climate and Environment, Ragnvald Blakstadsv. 1, Postbox 788 Stoa, 4809 Arendal, Norway. The permission was given to the Institute of Marine Research under the prerequisite that details on the catch were reported when the investigations were finished. The report was delivered to the authorities according to the plan. Our study did not involve endangered or protected species.

**Table 1:** Total number of herring caught in the local area for 2012, in brackets number of gillnets; ice = no sampling possible because the area was covered by ice.

Date	Landvikvannet	Inner Strandfjorden	Outer Strandfjorden	Bufjorden
15/2	Ice cover	Ice cover	28 (1)	11 (1)
6/3	4 (3)	129 (1)	119 (1)	
20/3	47 (3)	542 (1)		
26/3	115 (3)	486 (1)		100 (1)
11/4	290 (2)	663 (1)		
14/5	177 (1)	69 (1)		
21/6	82 (1)	66 (1)		
**Total**	**715**	**1955**	**147**	**111**

Biological samples were analyzed according to IMR standard protocols [46]. The maximum sample size was 100 herring. Biological parameters included in the present study were total length (nearest 0.5 cm below), weight (nearest gram below), sex, stage of maturity, age (otolith readings) and vertebral count (VS). Maturity stages were determined by visual inspection of gonads according to the following scale: immature = 1–2, maturing = 3–4, ripe = 5, spawning/running = 6, spent = 7 and recovering = 8 [46].

### Image and shape analyses

**Figure 2:**
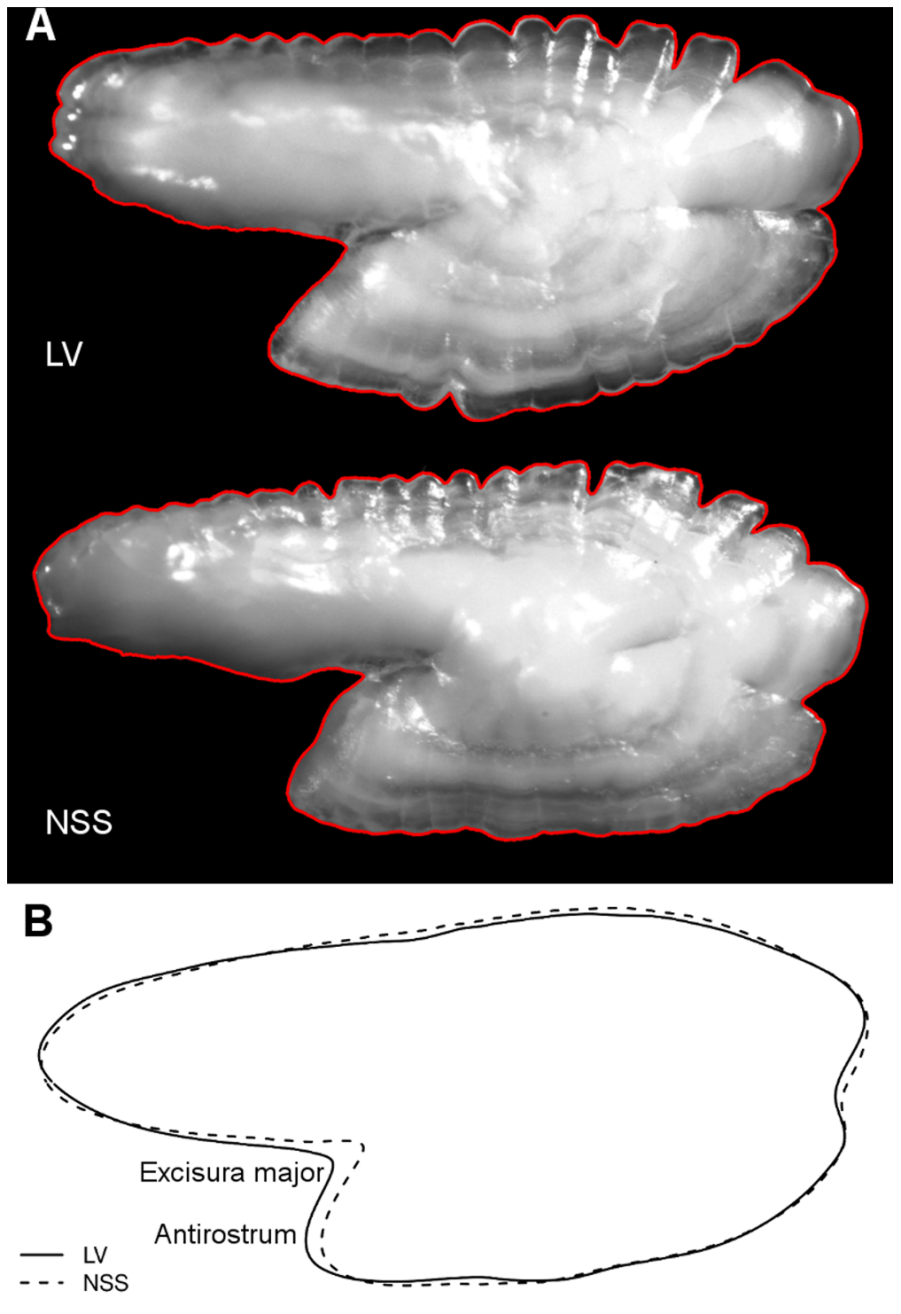
Example of otolith characteristics from two herring populations. A) Example of otoliths used for the shape analysis from Landvikvannet herring (LV) and Norwegian spring-spawning herring (NSS), both at the age of 3 years. Individuals of NSS herring were subjectively identified based on a sharper distinction between winter (dark areas) and summer rings (white areas). Red outline marks the shape of the otolith which was used to compare among populations. B) shows the mean shape of otoliths for the two populations, where the excisura major and antirostrum areas are the most variable areas.

Individuals of NSS herring were identified from otoliths, based on a sharper distinction between winter and summer rings compared to local spring spawners (Figure 2). This distinction was also independently tested using image and shape analyses of the otoliths. The rest of the individuals were divided into two populations based on sampling location: local Landvikvannet herring (LV) sampled inside Landvikvannet and coastal Skagerrak spring spawners (CSS) sampled outside Landvikvannet (Table 2). We expected that LV herring would mainly consist of individuals with similar biological characteristics as normally found in May, whereas the CSS herring would mainly consist of spring spawners with characteristics normally found along the Skagerrak coast during February–June. However, some mixture of the two populations would be expected, and this would be evident from results of the biological analyses. To investigate changes in the mixture of NSS, CSS and LV herring in the two habitats, selected biological characters (otolith shape, vertebral count, growth and maturation stage) were analyzed over the full season. The numbers analyzed by month and population are given in Table 2.

**Table 2:** Total number of herring analyzed in 2012 by month for the three putative herring populations, Norwegian spring spawners (NSS), Coastal Skagerrak spring spawners (CSS) and Landvik herring (LV), in brackets number of NSS inside Landvikvannet.

Month	NSS	CSS	LV
2	7 (0)	32	0
3	108 (38)	440	113
4	32 (14)	68	86
5	8 (5)	61	95
6	0 (0)	66	77
**Total**	**155 (57)**	**667**	**371**

Otolith shape was analyzed using the programming language R [47]. Outlines of otoliths were collected from digital images using the package pixmap [48], and applying the conte function [49] to record a matrix of X and Y coordinates (Figure 2a). Mean shape of otoliths differed among the populations, where the modifications in the shape of otoliths mainly were found at the excisura major and antirostrum areas (Figure 2b).

To remove size-induced bias, otolith sizes were standardized to equal area by dividing the coordinates of each otolith with the square root of the otolith area. Equally spaced radiis were drawn from the otolith centroid to the otolith outline, using the regular radius function [49]. Independent Wavelet shape coefficients were obtained by conducting a Discrete Wavelet transform on the equally spaced radiuses using the wavethresh package [50]. To determine the number of Wavelet coefficients needed for the analysis, the deviation of the reconstructed Wavelet otolith outline from the original outline was evaluated. To correct for fish length, an ANCOVA was performed on the wavelet coefficients taking fish length as a covariate. Coefficients which could not be adjusted by linear relationships on fish length, due to interaction between the origin and length were excluded from the analysis [51]–[53]. To adjust the Wavelet coefficients for allometric growth, a normalization technique based on regression was applied to scale the Wavelet coefficients [54].

### Data analyses

The number of gillnets varied between Landvikvannet and the neighboring fjord area. Therefore, to estimate the proportions of the LV, CSS and NSS herring, the total catches landed were standardized by catch per unit effort (CPUE), i.e. catch per gillnet.

All statistical analyses were conducted in R (version 3.0.1; [47]). A significance level of α = 0.05 was used for all statistical tests. For the plots, mean and standard error (1 SE) are shown. Some samples had very few or no data, and samples with N<5 were excluded.

Analysis of Covariance (ANCOVA) was used to test for sex differences in the biological characters (length, age, VS and stage of maturity). Differences in VS among different herring populations were assessed using Analysis of Variance (ANOVA), and a Kruskal-Wallis test for length and age variables as these were not normally distributed. For pairwise comparisons of VS a paired T-test was used, and the Mann-Whitney test for length and age comparisons.

Length-at-age data, used as a proxy for growth of individual herring, were fitted to the von Bertalanffy growth model (VBGM) [55]:
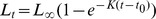
where *L_t_* is the average length at age *t*, *L_∞_* is the asymptotic maximum length, *K* is the von Bertalanffy growth rate coefficient, i.e. the rate at which length approaches the maximum length asymptote and *t_0_* is the intercept on the time axis. Growth was compared between the different groups using ANOVA.

Variation in otolith shape, as reflected by the scaled Wavelet coefficients, was analyzed with Canonical Analysis of Principal coordinates (CAP) [56] using the capscale function in the vegan package in R [57]. Using multivariate data to represent otolith shape, an ANOVA like permutation test (vegan package) was used to assess the significance of constraints using 5000 permutations. Variation in otolith shape was analyzed with CAP, while length and VS were compared with ANOVA with respect to herring group: NSS, LV and CSS, the month in which they were caught over the sampling period (Feb–June) and age in years (3–12) using the following models: shape∼herring population*month*age, length∼herring population*month*age and VS∼herring population*month*age. Non-significant interaction terms (p>0.05) were excluded from the models. *P*-values for all posteriori comparisons were corrected with the Bonferroni correction [58]. Possible trends of length and VS within herring populations were tested for significance using linear regression, while the stage of maturity was tested with the Spearman's rank correlation coefficient. For the comparisons of environmental data at time of spawning with the VS of herring, measurements from 3 m were used for Landvikvannet due to the depth of oxygen depletion in combination with previous (2010) acoustic observations of school depth [43]. In Strandfjorden, measurements from 5 m were used, based on acoustic observations of herring school depth during tagging experiments and the gillnet sampling [43].

## Results

### Environmental conditions

**Figure 3:**
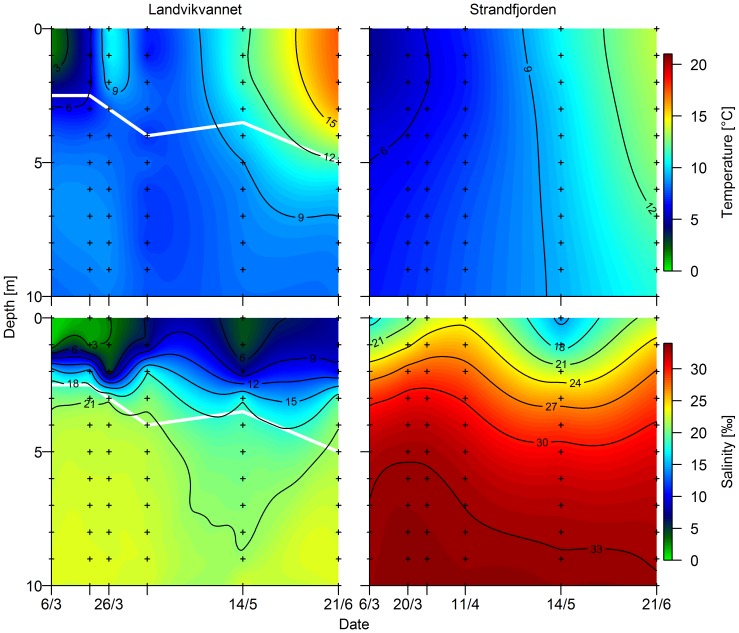
Seasonal change in temperature and salinity by depth. Temperature (upper) and salinity (lower) in Landvikvannet and in Strandfjorden over the study period from March to June. White line indicates the depth of oxygen depletion.

The environmental conditions differed considerably between Landvikvannet and the neighboring fjord, and changed over the spawning season in both locations (Figure 3). Anoxic conditions were found in Landvikvannet at increasing depths from 2.5 m in March to 5 m in June. Salinity ILV at 0–1 m increased over the season from 1‰ in March to 7‰ in June, but was stable around 20–25‰ deeper than 10 m. In comparison, there were no anoxic conditions in Strandfjorden, the salinity at 0–1 m increased from 10‰ in March to 25‰ in June and was stable at 35‰ deeper than 5 m. The temperature at 0–5 m depth increased from March to June from 5 to 17°C in Landvikvannet, and from 7 to 14°C in Strandfjorden.

### Population structure

A total of 1260 herring were analyzed during the 2012 spawning season. Total length ranged from 22.0–34.5 cm (mean: 28.3 cm) and age from 2–12 years (mean: 4.2 years). None of the biological characters varied between sexes (*p*>0.05). Hence, all further analyzes were carried out with sexes combined.

**Figure 4:**
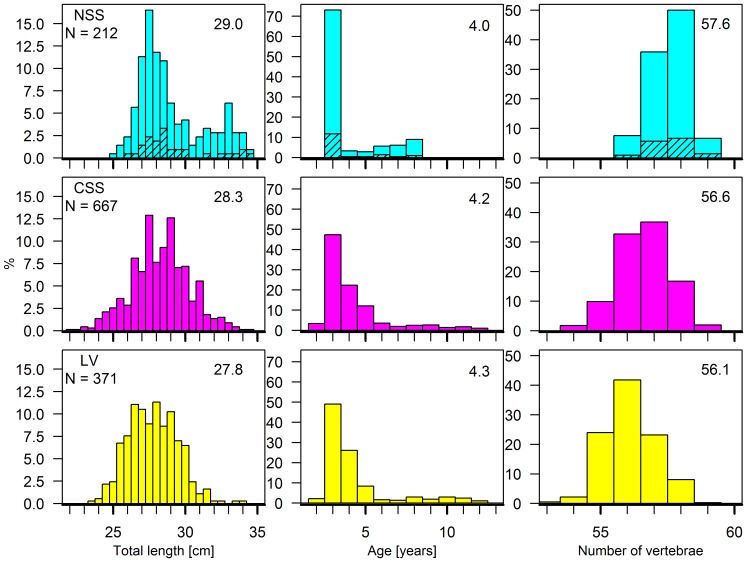
Distribution of length, age and vertebral counts of different herring populations. Comparison between Norwegian spring spawning (NSS), Coastal Skagerrak spring spawning (CSS) and Landvik (LV) herring. Shaded areas are NSS herring inside Landvikvannet. The mean values are included.

Mean length, age and vertebral count (VS) differed significantly among the three herring populations (*p*<0.001, Figure 4). For age and length, pairwise comparisons were also significant (*p*<0.001), with the exception of CSS versus LV for age (*p*>0.05). The vertebral count differed significantly (*p*<0.001) for all pairwise comparisons. The main tendency was a significant increase in mean body length and VS when moving from LV to CSS to NSS, whereas men age decreased. The most common age was 3 years for NSS, CSS and LV herring. The 4 year olds were also abundant in CSS and LV herring, but hardly present among NSS herring.

**Figure 5:**
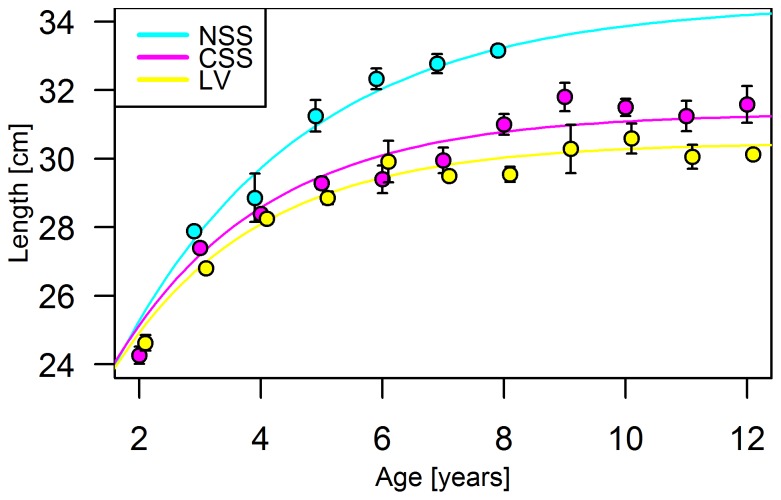
Growth curves of different herring populations. Length-at-age for Norwegian spring spawning (NSS, N = 212), Coastal Skagerrak spring spawning (CSS, N = 667) and Landvik (LV, N = 371) herring in samples pooled over the 2012 spawning season. Means and standard error (1 SE) are given, lines show van Bertalanffy growth models fitted to data.

Length-at-age data indicated the highest growth for NSS herring, and lowest for LV herring (*p*<0.01) (Figure 5). The von Bertalanffy growth model supported these growth differences (Table 3). Consequently, there were three categories: ‘high growth rate’ (NSS herring), ‘moderate growth rate’ (CSS herring) and ‘low growth rate’ (LV herring).

**Table 3:** Von Bertalanffy growth parameters (L_∞_, k, and t_0_) of herring populations Norwegian spring spawners (NSS), Coastal Skagerrak spring spawners (CSS) and Landvik herring (LV).

	L_∞_	K	t_0_
NSS	34.51	0.33	−1.98
CSS	31.31	0.41	−1.98
LV	30.33	0.43	−1.98

**Figure 6:**
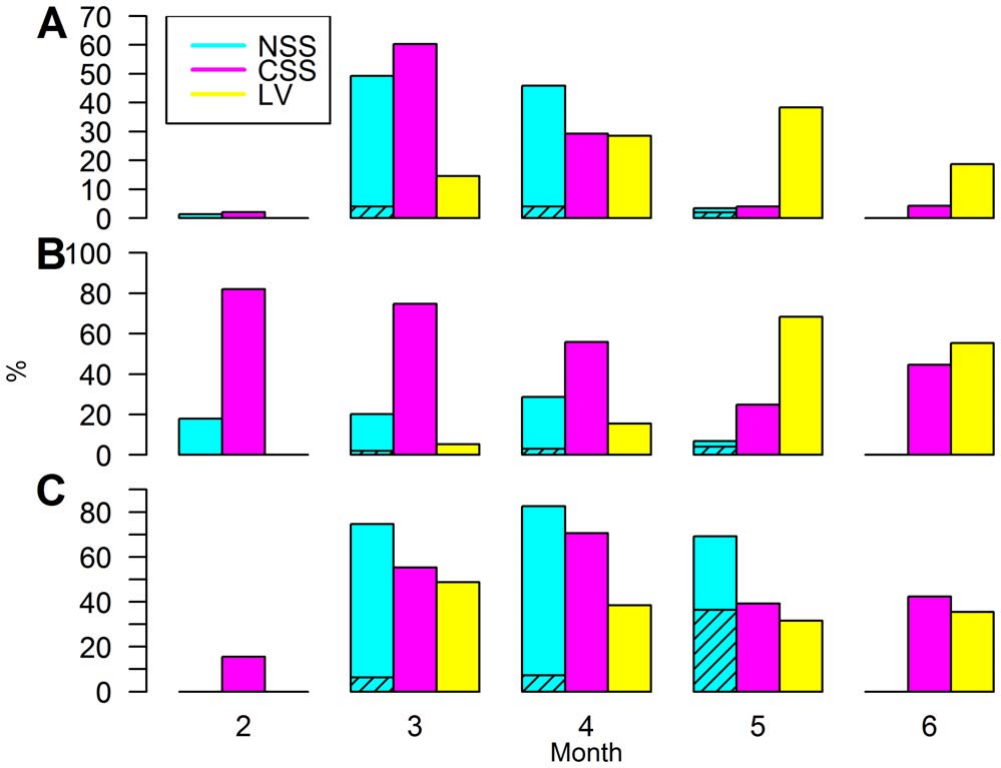
Seasonal change in proportion of different herring populations. Proportion (%), standardized to one gillnet per sample and area, by month of Norwegian spring spawning (NSS), Coastal Skagerrak spring spawning (CSS) and Landvik (LV) herring relative to a) total number analyzed over entire study period (see Table 1 for N), b) total number at month and c) spawning and spent herring (stage of maturity> = 6) relative to total number at month (see Table 2 for N). Shaded areas are NSS herring inside Landvikvannet.

Between February and June there was a change in the abundance of the different populations (Figure 6). During February–April CPUE was highest for CSS and NSS herring with a low proportion of LV herring (<20%). Also the proportion of NSS herring entering Landvikvannet was insignificant (<10%). The proportion of spawning and spent herring during this period was highest in NSS herring and a little lower for CSS herring, but still indicating peak spawning of two different populations in the fjord habitat during this period. Among the LV herring analyzed in March–April an even lower proportion were in spawning and spent stages than for CSS herring, indicating a later spawning peak for LV herring. This was further demonstrated in the May–June sampling showing a spatial shift in CPUE towards higher abundance of LV than CSS and NSS herring.

**Figure 7:**
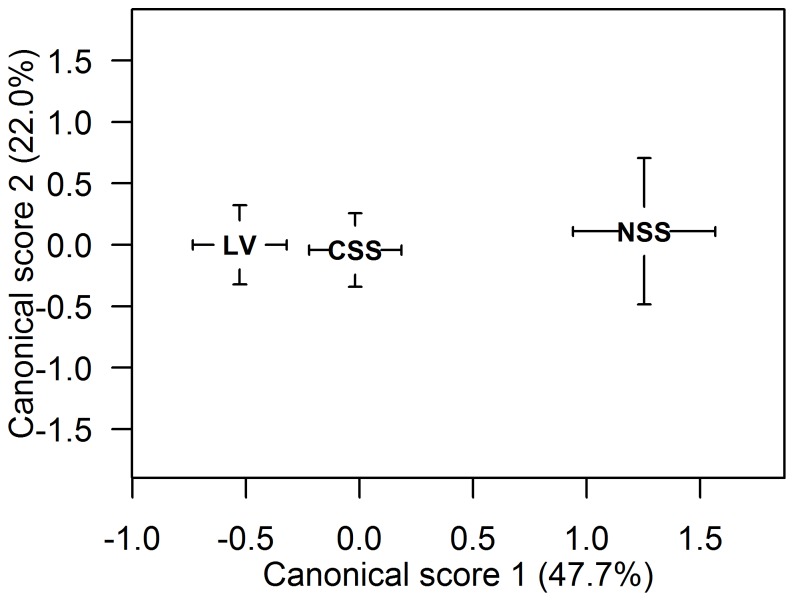
Otolith shape compared for different herring populations. Canonical scores for Norwegian spring spawning (NSS, N = 152), Coastal Skagerrak spring spawning (CSS, N = 397) and Landvik (LV, N = 348) herring are shown on discriminating axes 1 and 2. Black letters represent the mean canonical value for each group with standard error of the mean (1 SE).

**Figure 8:**
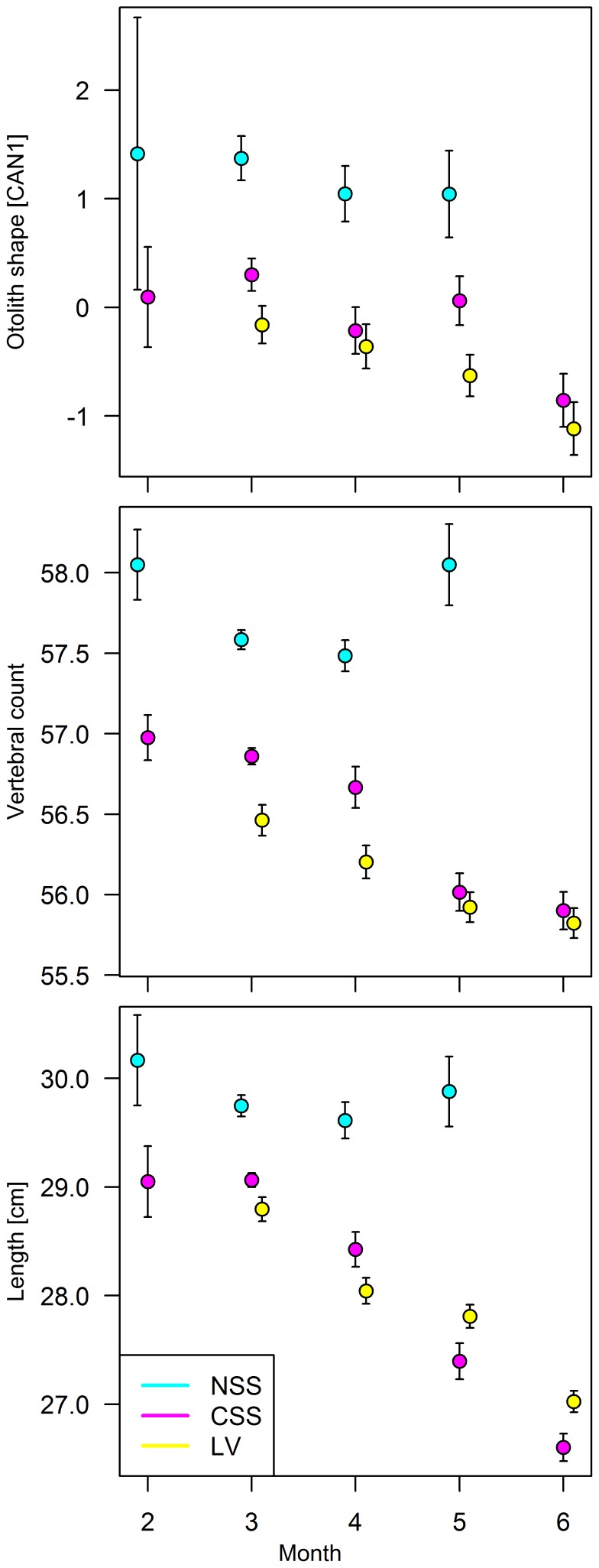
Seasonal changes of otolith shape, vertebral counts and length for different herring populations. For standardized ages. Comparison between Norwegian spring spawning (NSS), Coastal Skagerrak spring spawning (CSS) and Landvik (LV) herring (see Table 2 for N). Values given are means and standard errors (1 SE).

Otolith shape differed among the three herring populations (*p*<0.001, Table 4, Figure 7) and also varied though the spawning season (*p*<0.001, Figure 8A). Vertebral count and length differed between the populations (*p*<0.001) and between months (*p*<0.001, Figure 8B, C). Age was a significant factor for all characters (*p*<0.001) and therefore incorporated in the model for all comparisons. Posteriori comparisons showed that LV and CSS differed in otolith shape, VS and length (*p*<0.04, Figure 8, Table 4). NSS and LV (*p*<0.001) as well as NSS and CSS (*p*<0.02) also differed, while no differences were detected for NSS caught inside or outside the lake (*p*>0.05). There was a signifiant (*p*<0.001) negative trend in the mean Canonical scores (CAN1) derrived from the CAP analysis of otolith shape, vertebral count and length for LV and CSS herring at standardized ages over the spawning season, but not for NSS (Figure 8). This indicates that LV herring, characterized by slow growth and low vertebral count, were arriving and mixing with CSS herring.

**Table 4:** Comparing otolith shape, vertebral count (VS) and length among herring populations Norwegian spring spawners (NSS), Coastal Skagerrak spring spawners (CSS) and Landvik herring (LV).

			Otolith shape				Vertebral count				Fish length			
Comparison		N	df	Var	F	P	df	Mean Sq	F	P	df	Mean Sq	F	P
Overall	NSS vs LV vs CSS	897	2	3.28	5.36	<0.001	2	109.95	136.44	<0.001	2	129.80	102.58	<0.001
	Month		1	1.20	3.91	<0.001	1	71.49	88.71	<0.001	1	690.00	545.44	<0.001
	Age		10	4.49	1.47	0.001	10	3.87	4.80	<0.001	10	178.20	140.90	<0.001
	Residuals		883	270.41			867	0.81			867	1.30		
Posteriori	LV vs CSS	745	1	0.69	2.22	0.04	1	32.10	36.69	<0.001	1	13.10	10.08	0.006
	NSS vs LV	500	1	1.45	4.76	<0.001	1	219.80	276.99	<0.001	1	250.45	196.30	<0.001
	NSS vs CSS	549	1	0.84	2.72	0.02	1	115.53	149.39	<0.001	1	178.20	114.88	<0.001
	NSS-ILV vs NSS-OLV	152	1	0.20	0.65	>0.05	1	0.23	0.47	>0.05	1	1.85	1.65	>0.05

NSS herring were also compared between sampling locations, inside (NSS-ILV) and outside (NSS-OLV) Landvikvannet. ANOVA like permutation tests were used to assess the difference in otolith shape and ANOVA for the vertebral count and fish length comparisons. For otolith shape: df: degrees of freedom, Var: Variance among populations, F: pseudo F-value, P: proportion of permutations which gave as large or larger F-value than the observed one. For the vertebral count and fish length: df: degrees of freedom, Mean Sq: Mean Square, F: F-value, P: P-value. P-values for posteriori comparisons have been corrected with a Bonferroni correction. P<0.05 indicates a significant effect.

### Maturation and spawning time

Herring in spawning condition were present and overlapped in time for LV, CSS and NSS herring, however, maturation and timing of spawning was delayed in LV compared to NSS and CSS herring (Figure 6). This indicates an adaptation to the environmental conditions and seasonal change in Landvikvannet. Since differences in vertebral count are linked to environmental conditions, the temperature and salinity at depth and time of spawning affects the vertebral count. The salinity at expected spawning depth in Landvikvannet was distinctly lower (10–15‰) than in the adjacent fjord (>30‰), which could explain the low vertebral count observed in Landvikvannet. The vertebral count was not significantly related to change in salinity over season within habitats; there was negligible change at assumed spawning depth. However, there were significant changes in temperature over season in both habitats, coinciding with a significant decrease in vertebral count at spawning time for both CSS and LV herring (*p*<0.05).

## Discussion

This study reveals strong seasonal dynamics involving three populations of a pelagic migratory fish, the Atlantic herring, in the vicinity of a marginal inland brackish water lake habitat (Landvikvannet) on the Norwegian Skagerrak coast. Gillnet sampling was standardized, implying that the observed differences between herring populations and over season dynamics were not affected by the selectivity normally experienced with gillnet sampling [59]. Three putative herring populations were identified; Norwegian spring spawners (NSS), Landvik herring (LV) and Coastal Skagerrak spring spawners (CSS). Individual NSS herring were identified subjectively based on otolith growth characteristics, and statistically based on otolith shape and mean vertebral count (57.5). NSS herring also had higher growth than the other populations, which is typical for this stock [13], [43]. Identification of individual CSS and Landvik herring was not possible. Individuals sampled inside the lake were all classified as LV herring, whereas those sampled outside the channel connecting the lake to the sea were assigned as CSS herring. However, there was a significant decrease in vertebral count over the sampling season in both LV and CSS herring, from levels known as typical for CSS herring (56.5–56.9) in March–April to levels typical for Landvik herring (<56.0) in May–June, again based on historic data [43]. This trend in vertebral count was followed by a decrease in size and change in otolith shape, and a marked change in the relative proportions of the two populations.

The observed seasonal dynamics in biological characters clearly indicate that the assignment of individual fish into CSS and LV herring simply based on sampling location was uncertain, and that the two populations were mixing both inside and outside the lake habitat together with NSS herring showing a different peak occurrence. Early in the season in February–April the biological characteristics indicated that NSS and CSS herring predominated, with only small numbers entering the lake. There was a clear temporal and spatial overlap in spawning individuals from these two populations, although proportions spawning in CSS were comparatively lower than in NSS herring. In May–June there was a significant change with the appearance of a new spawning wave of LV herring, with the highest proportion found inside the lake. Still, the immigration of this population was evident throughout both habitats, where many of the herring found in the fjord would be expected to enter the lake. The data on otolith shape, vertebral count and growth in May tended to differ from the observations in June in both locations, which indicated a spatial and temporal overlap in May between minor proportions of NSS and CSS herring completing their spawning season at the same time as the LV herring was peaking.

All three putative populations were caught at the same location, in the same gillnets, at the same time with running gonads, suggesting that the populations together form a metapopulation [40]. However, there is doubt as to whether interbreeding between distinct populations is occurring despite their proximity in spawning condition. Since breeding was not observed directly, one cannot exclude the possibility that the populations separate for spawning events. Such a full separation seems unlikely for NSS and CSS herring because of the high temporal and spatial overlap; whereas it seems more likely for LV herring considering the limited temporal and spatial overlap with the other populations.

The idea that LV herring is reproductively isolated from other populations may be supported by the low vertebral count and concept of natal homing. Differences in vertebral count stem from the incubation phase and thus reflect the origin of the fish at spawning [60]. In general, there is a positive correlation with salinity [31] and negative with temperature [21], [29], [61] experienced prior to hatching. Hence, the warmer and less saline ambient environment for herring occurring inside Landvikvannet in May–June compared with that experienced by CSS in March–April in the fjord habitat, could result in the observed differences in vertebral count. The low vertebral count of LV herring and the late timing of spawning is an indication of spawning and adaptations to the environmental conditions of the lake habitat. However, this also implies that natal homing [62], [63] of Landvik herring occurs on an annual basis. The vertebral number for LV herring in May has been remarkably stable (55.5–55.8) since 1984 [43], supporting natal homing. The principle of natal homing is central to the discrete population concept [12]. Moreover, recent genetic studies support the occurrence of natal homing of herring in the North and Baltic Seas [6], [64]. Likewise, Brophy et al. [65] suggested that spawning season and location of Atlantic herring could be predetermined and not learnt from repeated spawning [66]. Support for natal homing and adaptations of Landvik herring to environmental conditions of its marginal habitat also originates from a recent genetic study using 20 microsatellite markers, where Landvikvannet differed from other local herring in Lindåspollene, Lusterfjord and Trondheimsfjord as well as from other herring populations surrounding the Norwegian Sea [67]. Unpublished results on the microsatellite locus Cpa112, which is non-neutral to salinity variability with allele frequencies varying from 45% in the Baltic to 2–4% in the North Sea [27], have shown that Landvik herring is obvious with a frequency of 15% (Carl André, pers. Comm., Department of Biology and Environmental Sciences - Tjärnö, University of Gothenburg, Strömstad, Sweden).

It seems clear from this study that we can refute the hypothesis of a resident local population inside the lake; LV herring definitely migrates into the lake habitat from coastal areas. In this sense the Landvik herring differs from other local herring populations, such as the Trondheimsfjord or Lindås herring, which can be observed throughout the year in their local areas [32], [33], [36], [41]. This may simply be because of the unsuitability of this location as a nursery area for juveniles and feeding grounds for adults. Both CSS and LV herring may still represent more stationary coastal populations not undertaking large scale oceanic migrations. The observed relatively low investment costs in reproduction (low GSI) of NSS compared with that of LV herring supports the assumption that NSS is more migratory [44]. The fact that growth of CSS was higher than in LV herring, further suggest that these two populations may not overlap much during the nursery period or at adult feeding grounds. In fact, there is probably little or no spatial overlap for most of the year, with overlap only occurring during the spawning season.

The movements of herring between the fjord and Landvikvannet habitats have also been studied with acoustic telemetry [43], [68]. The telemetry study showed that some fish moved in and out of the lake habitat, whereas others stayed inside the lake for more than two weeks. Those fish that arrived and only stayed for a short period of time were interpreted as being NSS or CSS, whereas the ones remaining in the area for extended periods of time were thought to be local LV herring. It is likely that some NSS and CSS herring have short visits to the lake as exploratory migrations searching for good habitats cued by the current from the Reddal channel, but migrate out again to spawn in areas which are more characteristic of their normal spawning habitat. Conversely, fish that stay for two weeks inside the lake before leaving is a reasonably good indication of an established adaptation to the lake and to potential spawning within the lake.

The appearance of NSS herring in the habitats within Landvikvannet and adjacent fjords probably does not represent natal homing. The predominance of 3-year-olds among the NSS stock as well as the high stability of growth and meristic characters over the season, suggest independent selection of spawning grounds, as supported by Slotte and Fiksen [69]. In NSS herring specifically, the use of spawning grounds other than their natal ground is common. NSS herring have a tendency to change their spawning ground as they grow older with larger fish tending to migrate further, in this case southward, and thus potentially increase their life time fitness [69]–[71]. Such straying from natal spawning grounds results in considerable gene flow [72], [73]. The predominance of 3-year-old NSS mixing with CSS and Landvik herring in 2012 may be explained by the relatively unusual spawning migrations of NSS herring in 2009–2010. During these two years a significant proportion of the adult NSS migrated from wintering grounds in the northern Norwegian Sea to areas south of 60°N, resulting in the largest fishery in the fjords (e.g. Boknafjorden) east of the traditional spawning grounds off Karmøy since the 1950s [74]. Based on vertebral count and growth data, it was apparent that the fishery was targeting NSS herring [75] and the abundance was high as evaluated by catch levels (Table 5). One hypothesis is that the 3 year old NSS mixing with CSS and Landvik herring in 2012 was a result of this significant spawning at the southern grounds in 2009. Generally, if first time spawners of NSS do not meet older conspecifics and learn to follow their migration towards the spawning grounds then the location of the spawning ground is a chance event [70], [71], [76], [77]. In addition, NSS herring tend to migrate upstream to spawn [69]. Therefore it is not unlikely that NSS from Boknafjorden or further south may have spawned close to their nursery areas or even migrated further south-eastwards against the coastal current to spawn. In addition, school composition tends to involve size-matching among individuals [78], in this case younger, smaller NSS. Three year old NSS (mostly first-time spawners), may have adopted the behavior of the joint local populations with whom they mix during the nursery period as postulated in the adopted-migrant hypothesis [40], [79].

**Table 5:** Commercial catches of herring off Karmøy 2005–2012.

	Year of catch							
Month	2005	2006	2007	2008	2009	2010	2011	2012
1					0.1			
2	21.2				172.0	3302.9	609.1	897.3
3	24.5	32.6	16.5		19052.0	14877.0	6528.4	6283.2
4	129.2	0.7	1.0	4.8	2301.2	1000.3	52.0	13.4
8	1.0							
9					0.9			
10			0.1					
11		72.8				0.5		
12	0.2							
**Total**	**176.1**	**106.1**	**17.6**	**4.8**	**21526.2**	**19180.7**	**7189.5**	**7193.9**

Live weight (tons) calculated from landed weight to live weight equivalent for Norwegian spring spawning herring in the Norwegian statistical area 08 (SW coastal Norway) by month and year as registered in the Directorate of Fisheries database.

**Figure 9:**
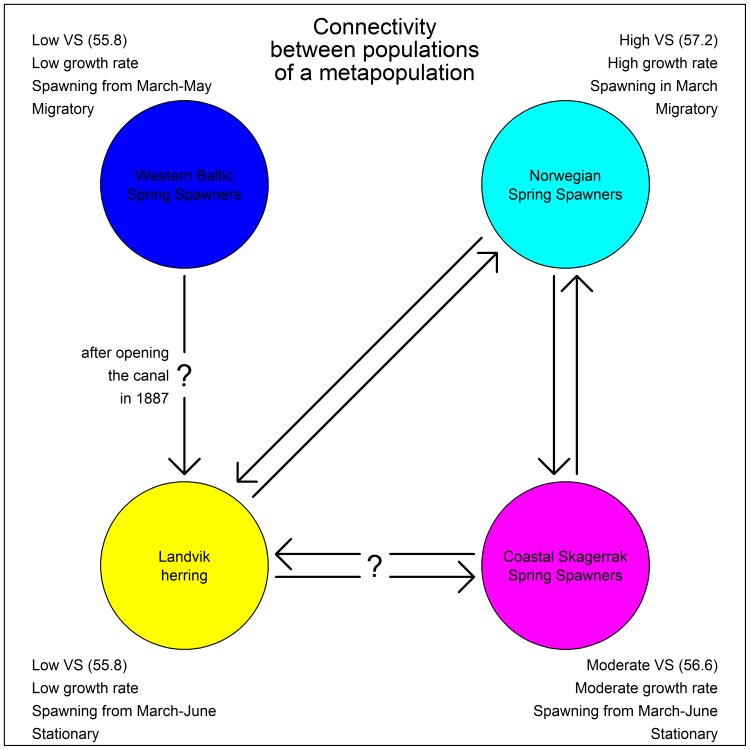
A schematic model of potential metapopulation dynamics in the study area. Potential connectivity between populations of a metapopulation in the study area of Landvikvannet and the connected fjords as hypothesized based on the results of the present study. The biological characteristics (VS = vertebral counts) of the different populations are given.

From an evolutionary perspective, the Landvikvannet habitat has only been available for marine species for a relatively short period of time. This raises the question of the origin of the herring first colonizing the lake after the opening of the Reddal channel (Figure 9). One possibility is that CSS herring entered the lake sometime after the opening of the channel and successfully spawned there. Due to lower salinity and higher temperature in the lake the offspring developed significantly divergent characters over the years. A strong natal homing effect of herring would lead to the development of a new local population inside Landvikvannet. Hendry and Kinnison [80] concluded that a time span less than 100 years can be sufficient for significant microevolution to develop in response to local agents of selection. Also, Neb [81] demonstrates that such a time interval and differences in salinity are sufficient for herring to diverge in meristic characters. This explanation assumes reproductive isolation during spawning between the original CSS herring and the “new” Landvik herring. A second possibility is that the origin of Landvik herring could be Western Baltic Spring Spawners (WBSS) herring. First time, or even repeated, spawners could have established a new spawning ground in Landvikvannet. The reason for not conducting an annual migration to the original spawning grounds off the island Rügen may be a trade-off between survival of progeny and physiological migration constraints, as shown for NSS by Slotte [70]. WBSS close to their feeding grounds in the Skagerrak could have “discovered” Landvikvannet, cued by similar environmental conditions as those of their original spawning grounds. The continued link to Landvikvannet may have been a result of a fidelity to this site rather than for joining conspecifics in a migration back in to the Baltic region. Huse et al. [76] demonstrate that a high ratio of first-time spawners could lead to the establishment of new wintering grounds. In the case of Landvik herring, it may have led to a new spawning ground.

In conclusion, the present study provides evidence for a distinct small local population of herring associated with Landvikvannet, partly mixing with NSS and CSS herring. This population of LV herring resides, during part of the year in brackish water with many morphometric characteristics indicative of spawning in warm and low salinity environments. Whilst ripe and spent fish have been found in the area, there is no direct evidence of spawning in the lake. If spawning does occur there are no data to indicate likely survival rates or even the residence time of offspring in the lake. There has been one attempt to find eggs with a diver for 1 hour at one of the many bays in the lake, without success. Also, limited plankton net sampling in selected parts of the lake have failed to capture any larvae. The only evidence of potential spawning in the lake, is from two eels with stomachs full of fertilized herring eggs. There is also no clear evidence of the origin of this population, however, they could have arisen from either WBSS or other local CSS. The presence of mixtures of these and other stocks and populations in the Skagerrak area have been shown previously [6], [82]. Recent genetic studies using microsatellite DNA [83] have demonstrated differences between Landvik herring and many other stocks, in addition, unpublished results on one microsatellite locus (Carl André, pers. Comm., Department of Biology and Environmental Sciences - Tjärnö, University of Gothenburg, Strömstad, Sweden) suggesting that Landvikvannet herring has not recently immigrated from the Baltic.

The results of the present study may also have some implications for the official ICES stock assessment of herring in the North Sea and Skagerrak area. The present work demonstrates that there can be a fairly complex population structure in the areas with more than one ‘stock’ which can be mixed. Whilst this may not be a significant problem for the assessment of NSAS or WBSS due to the relatively small abundances of CSS and LV herring, there is a possibility that these smaller populations could be very vulnerable to overfishing [9]. This is probably not unique for coastal areas as there are a number of relatively small populations bordering the North Sea and Skagerrak area [84].

From management point of view, probably the most striking result of the present study is the conclusive evidence of NSS herring as far southeast as in the Skagerrak. This is the first time that individuals from this historically large herring stock have been studied in the Skagerrak area. By definition this stock is not exploited south of 62°N, with exception of the spawning period when they previously have been found as far south as to Lindesnes (Figure 1). This signifies that migration dynamics and population connectivity among herring in the Northeastern Atlantic may be more dynamic than previously assumed, and this must be taken into account in the future development and implementation of new management strategies.
